# The Cost of Cost-Sharing: The Impact of Medicaid Benefit Design on Influenza Vaccination Uptake

**DOI:** 10.3390/vaccines5010008

**Published:** 2017-03-06

**Authors:** Charles Stoecker, Alexandra M. Stewart, Megan C. Lindley

**Affiliations:** 1 Department of Global Health Systems and Development, Tulane University School of Public Health and Tropical Medicine, New Orleans, LA 70118, USA; 2 Department of Health Policy, The George Washington University School of Public Health and Health Services, Washington, DC 20052, USA; stewarta@gwu.edu; 3 National Center for Immunization and Respiratory Diseases, Centers for Disease Control and Prevention, Atlanta, GA 30333, USA; cvx9@cdc.gov

**Keywords:** vaccination, Medicaid, influenza

## Abstract

Prior research indicates that cost-sharing and lack of insurance coverage reduce preventive services use among low-income persons. State Medicaid policy may affect the uptake of recommended adult vaccinations. We examined the impact of three aspects of Medicaid benefit design (coverage for vaccines, prohibiting cost-sharing, and copayment amounts) on vaccine uptake in the fee-for-service Medicaid population 19–64 years old. We combined previously published reports to obtain state Medicaid policy information from 2003 and 2012. Data on influenza vaccination uptake were taken from the Behavioral Risk Factor Surveillance System. We used a differences-in-differences framework, controlling for national trends and state differences, to estimate the effect of each benefit design factor on vaccination uptake in different Medicaid-eligible populations. Each additional dollar of copayment for vaccination decreased influenza vaccination coverage 1–6 percentage points. The effects of covering vaccines or prohibiting cost-sharing were mixed. Imposing copayments for vaccination is associated with lower vaccination coverage. These findings have implications for the implementation of Medicaid expansion in states that currently impose copayments.

## 1. Introduction

It is well-documented that having health insurance coverage is associated with greater likelihood of receiving recommended adult vaccines. This finding holds true across a range of adult subpopulations and vaccine types [1,2,3,4]. Few studies examine the effect of insurance coverage for specific preventive services, although one study using self-reported data found that insurance coverage for vaccinations was associated with increased vaccination uptake [3]. Prior research also shows that cost-sharing reduces use of medical care, including recommended preventive care [5], and that the resulting reductions in usage may disproportionately affect health outcomes for low-income individuals in poor health [6]. Studies have linked decreased out of pocket vaccination costs to increased immunization rates particularly for disadvantaged populations [7,8]. As a result, prior to the passage of the Affordable Care Act (ACA), several private health insurance plans waived copayments for vaccination services [9].

The Medicaid program was established in 1965 to provide health care coverage for low-income Americans and is one of the United States’ largest health care payers, serving close to 60 million people each year. The program is jointly funded by federal and state governments: the federal government’s share of every dollar spent varies by state based on the state’s per capita income, and is greater for states with lower per-capita incomes. States may choose to cover a variety of adults under their Medicaid programs; however, in order to receive federal matching funds, all states must cover certain low-income adult populations including: families with children and pregnant women [10]. These two groups are the focus of our analysis.

While the federal government establishes certain mandatory benefits, each state administers its own program and has considerable latitude to determine which optional benefits are covered for their adult beneficiaries. Similarly, the federal government places restrictions on the use of cost-sharing for certain Medicaid beneficiaries and services: for example, pregnant women are exempted from Medicaid cost-sharing requirements for pregnancy related services. However, within federal guidelines, states may choose whether to impose cost-sharing on other Medicaid services, such as vaccination, and what level of cost-sharing to impose [10].

Adult immunizations are an optional benefit for traditionally-eligible Medicaid beneficiaries, i.e., those who were eligible for Medicaid in their state prior to the passage of the ACA in 2010. In states choosing to expand their Medicaid programs in accordance with the ACA, all vaccines recommended by the Advisory Committee on Immunization Practices (ACIP) must be covered without cost-sharing for all newly-eligible adult beneficiaries. Therefore, it is possible that a substantial proportion of traditionally Medicaid eligible beneficiaries (who are the poorest and potentially most vulnerable), even in states expanding their Medicaid programs, may not have health insurance coverage that includes all vaccines routinely recommended for adults by ACIP.

In this paper, we examine the effect of copayment charges and other related elements of vaccination benefit design on influenza vaccination coverage among low-income adults. We use secondary survey data in a differences-in-differences framework to answer this question. These findings could potentially inform state-level Medicaid benefit design policy.

## 2. Methods

### 2.1. Data

Data on vaccine coverage policy were derived from two previous analyses of Medicaid vaccine benefit design [11,12]. These reports examined Medicaid benefit plans in 2003 and 2012 and used document review and a survey of state Medicaid administrators to categorize elements of Medicaid vaccine benefit design in fee-for-service Medicaid plans. Specifically, we drew from these reports three variables related to Medicaid vaccine benefit design: (1) whether the state covered influenza vaccines for traditionally eligible Medicaid beneficiaries; (2) whether the state prohibited cost-sharing for vaccination services provided to adult Medicaid beneficiaries; and (3) the copayment amount charged. Where cost sharing for immunization services was not permitted, we coded the copayment amount as zero dollars. We limited analysis for the impacts of cost-sharing and copayment amounts to states that covered influenza vaccine in both periods. We further restricted the copayment analysis to states that specified copayment amounts.

Data on vaccination coverage came from the Behavioral Risk Factor Surveillance System (BRFSS). The BRFSS is a nationally representative telephone survey of the non-institutionalized population that asks respondents whether they have received influenza vaccines. In 2003, the BRFSS asked respondents “During the past 12 months, have you had a flu shot?” For the 2012 BRFSS survey, the influenza vaccine question was reworded slightly to account for the nasal spray influenza vaccine which was first introduced in 2003. The question in 2012 read, “During the past 12 months, have you had either a seasonal flu shot or a seasonal flu vaccine that was sprayed in your nose?” The vaccination coverage levels presented in this analysis are based on reported vaccination in a calendar year and are not directly comparable to published estimates of seasonal influenza vaccination from BRFSS or other sources [13].

The BRFSS survey did not track Medicaid status, but recorded insurance status, state of residence, household income, number of people in the household, ages of household members, employment status, and pregnancy status. The pregnancy query asked, “To your knowledge, are you now pregnant?” We used these data to calculate the Federal Poverty Level for each respondent and matched this to state-specific income eligibility limits for different Medicaid populations (namely, in our study, pregnant women and parents) [14,15]. We limit our analysis to adults age 19 to 64, since nearly all adults aged 65 and over are eligible for Medicare, and Medicaid is a payer of last resort. The BRFSS does not track insurance status by source, but asks, “Do you have any kind of health care coverage?” Our imputed Medicaid populations excluded those that responded no, but included those that responded yes, did not know, or refused to answer the question. Therefore, our analysis population may include adults who have private insurance coverage in addition to Medicaid. Since Medicaid status was imputed based on other survey responses, we present results separately for all adults and for the imputed Medicaid populations.

Pregnant women and other adults have separate eligibility thresholds for Medicaid; therefore, we present results separately for these groups. We defined three groups of adults from the BRFSS data: (1) all adults; (2) Medicaid enrolled pregnant women; and (3) Medicaid enrolled parents. “All adults” included persons 19–64 years old regardless of income (i.e., Medicaid eligibility). Survey respondents were defined as Medicaid enrolled if the midpoint of the reported household income range was below the state limit for Medicaid eligibility for the particular qualifying category (pregnant, working parent, or nonworking parent), they reported having health insurance coverage, and they fell into one of the Medicaid eligibility categories included in our analysis. “Pregnant women” included female survey respondents who reported being pregnant at the time of the survey. “Parents” included respondents of either gender who reported there were children living in the household. While the 2012 BRFSS questionnaire included inquiries about relationship between the respondent and other household members, this was not asked in the 2003 survey.

Our analysis is limited to 49 states as no data were collected for the District of Columbia for 2003. Further, as Florida only covers vaccines for institutionalized Medicaid enrollees, and the BRFSS is limited to the non-institutionalized population, we coded Florida as not covering influenza vaccine and omitted it from the cost-sharing and copayment analyses.

Results in Table 1, Table 2, Table 3 and Table 4 are for respondents we impute to be insured with Medicaid based on the methods described above. We have explored alternative specifications that looked at Medicaid eligibility instead of Medicaid status and the conclusions were qualitatively unchanged. We have also explored specifications that combine identified categories of Medicaid eligible adults (parents and pregnant women) to increase the sample size, but again results were qualitatively unchanged.

We obtained the share of each state Medicaid population covered under fee-for-service Medicaid from the Kaiser Family Foundation [16].

### 2.2. Identification Strategy

We used a differences-in-differences approach [17] to examine the impact of state Medicaid benefits policies on vaccination coverage. Trends in states with no benefit changes were used as controls for states with benefit changes. We used year fixed effects to control for secular trends in vaccination coverage at the national level, and state-specific fixed effects to control for time-invariant state-level differences in vaccine uptake and the characteristics of each state’s Medicaid population, since the groups covered by Medicaid differ widely from state to state. Year fixed effects will capture any variation introduced at the national level introduced by changes such as the expansion of the populations recommended to receive influenza vaccination to include adults age 19–49 or the introduction of the flu nasal spray as a method of immunization. We tested our model’s robustness to this control by estimating an alternative specification of our model that includes only adults that were universally recommended to receive influenza vaccination in both 2003 and 2012. Results were estimated with a linear probability model that accounted for BRFSS’s complex survey sampling design and are weighted by the fee-for-service Medicaid population. All analysis was performed with Stata 13 (StataCorp, College Station, TX, USA) [18]. Institutional Review Board approval was not necessary as this study comprised secondary analysis of publicly-available data.

When performing our analysis of vaccination levels, we used the state-year as the level of analysis with population weights. We examined the effect of the dollar amount of any copayment for immunization service, whether the state Medicaid program prohibited cost-sharing, and whether the state Medicaid program covered the vaccine. Since our policy data were composed of two annual snapshots, one in 2003 and one in 2012, we were unable to determine exactly when during the nine years a state might have changed its policy. To account for this, we focused on the absolute change in vaccination uptake between 2003 and 2012.

The benefit design aspects examined in this study pertain to fee-for-service Medicaid. In order to account for varying levels of penetration of Medicaid managed care [19], we weighted results by the share of the state Medicaid population in fee-for-service plans.

## 3. Results

Our imputed estimates of Medicaid eligible populations using the BRFSS data were 14.0 million in 2003 (13.3 million parents and 1.0 million pregnant women) and 11.1 million in 2012 (10.6 million parents and 0.7 million pregnant women). This decline was driven by declines in eligibility levels for working or non-working adults in 37 states. These decreases in eligibility levels for adults were often combined with increases in eligibility levels for children—who are excluded from this study as they do not appear in the BRFSS and their vaccine and administration costs are covered under the Vaccines for Children Program. The weighted mean proportion of respondents reporting influenza vaccination increased slightly from 2003 to 2012 from 33.1% to 34.6% (*p* < 0.01) (Table 1). The proportion of state Medicaid programs prohibiting cost-sharing increased from 0% to 24.6%, and the mean copayment for Medicaid vaccination services decreased from USD$1.45 in 2003 to USD$1.22 in 2012. The proportion of state Medicaid programs providing coverage for influenza vaccinations for adult beneficiaries increased slightly from 2003 to 2012; however, most states already covered the vaccine in 2003.

Providing Medicaid coverage benefits for influenza vaccine led to a statistically significant increase in coverage levels for all adults (Table 2). Living in a state whose Medicaid program covered influenza vaccine was associated with a 3.6 (*p* < 0.001) percentage point higher likelihood of reporting being vaccinated. This estimate increased to 6.2 (*p* < 0.001) percentage points when we limited the sample to adults age 50–64 who were universally recommended to receive the influenza vaccine in both 2003 and 2012. The point estimate of the effect of covering the influenza vaccine was similar for pregnant women imputed to be enrolled in Medicaid (4.7 percentage points), but not statistically significant at the 5% level (*p* = 0.71). The estimated impact of covering the influenza vaccine on uptake in the Medicaid enrolled parent population was negative, but also not statistically significant.

The impact of prohibiting cost-sharing on influenza vaccination coverage was not statistically significant at the 5% level in each of the analytic groups (Table 3).

In our differences-in-differences model, Medicaid copayment charges negatively impacted influenza vaccination levels in each of the groups examined (Table 4). For all adults taken together, a one dollar increase in Medicaid copayments charged led to a 0.6 (*p* = 0.014) percentage point decline in influenza vaccination coverage. When the analysis was refined to include only adults age 50–64, the impact was larger at −1.1 (*p* = 0.014) percentage points. When we estimated this effect on our populations who were imputed to be on Medicaid, the point estimates were much larger (−6.2 for pregnant women and −1.4 for parents), but the 95% confidence interval for both of these estimates overlapped zero.

## 4. Discussion

Our study was one of the first to examine how vaccination coverage levels respond to financial incentives for Medicaid beneficiaries. Our results showed benefit policies that may impose financial barriers led to lower vaccination coverage levels among all adults. Of the three policy parameters we analyzed, we found that providing benefits coverage for influenza vaccine had the strongest effect on vaccination coverage. We also detected statistically significant decreases in influenza vaccine coverage among all adults when states charged larger copays. We found that the impact of covering the influenza vaccine on vaccine uptake was approximately equal in magnitude to (though opposite in sign of) charging an additional USD$5 copayment.

After imputing Medicaid enrollment status, we did not find statistically significant impacts of any of the three benefit policy variables on vaccine uptake in the enrolled subpopulations. One possible reason we detected impacts of Medicaid policies among the general population is that our “all adults” category included many adults who were actually eligible for Medicaid. We were unable to ascertain if these adults would have been eligible for Medicaid through mechanisms other than family income tests, or whose reported income was subject to measurement error.

Estimates of the impact of different vaccination benefit policies in Medicaid were highest for Medicaid-enrolled pregnant women in all cases, although none of these estimates were statistically significant. The larger point estimates for the Medicaid-enrolled pregnant women may indicate that they are more sensitive to additional financial barriers. Though Medicaid prohibits cost-sharing for pregnancy-related services; whether to permit cost-sharing for other services provided to pregnant women, including vaccination, is at the discretion of each state program [20].

Our inability to obtain more precise statistical estimates for the population of Medicaid-enrolled parents may be partly due to our inability to identify the legal parents in households where several adults live with children. BRFSS interviews only one adult per household and the relationship of children in the household to the interviewed adult was not assessed in 2003. Additionally, many of our estimates for pregnant women and parents may not have been statistically significant due to small sample sizes.

The major limitation of this study is that our data do not provide a direct indication of Medicaid eligibility or enrollment; instead we imputed Medicaid enrollment based on income and family size. The Medicaid eligible population age 19–64 was 21.3 million in 2003 [21]. The total number of Medicaid enrollees we imputed from the 2003 BRFSS was only 4.5 million, indicating imputing Medicaid eligibility based on income and household status in the BRFSS leads to identifying only a small fraction of eligibles. In 2009, 5% of Medicaid beneficiaries were “medically needy” [22], meaning that their income exceeded the Medicaid eligibility ceiling in their state of residence, but their medical expenses consumed a significant proportion of their income, rendering them eligible for Medicaid. Other categories of low-income adults eligible for Medicaid include recipients of Supplemental Security Income and blind or disabled persons. We were unable to identify these types of beneficiaries using the data available. Since Medicaid eligibility is measured with error, our results are likely biased toward zero and may underestimate the true negative impact of copayments and cost-sharing on vaccination coverage. Similarly, we may underestimate the true positive impact of covering a vaccine in the state Medicaid program on vaccination coverage. We were unable to obtain historical data on Medicaid income limits for non-parents who were not pregnant. This third group is included in the “all adults” category and may contribute to the direction of the associated point estimates. A related limitation is that pregnancy status is measured at the time of the survey, and income limits for Medicaid eligibility are higher (less restrictive) for pregnant women. Women imputed to be eligible for Medicaid at the time of the survey using pregnancy income limits may have not been pregnant at the time of influenza vaccination and thus been ineligible for Medicaid when the vaccine was received.

In addition to measurement error in Medicaid enrollment status, another limitation of this analysis is that vaccination status in the BRFSS is self-reported. While self-reported influenza vaccination status generally has high sensitivity (around 90%), it has a fairly low specificity around 50% [23,24]. Together, these mean that while vaccinated individuals generally reported being vaccinated, many unvaccinated individuals also reported being vaccinated. We note that this additional source of measurement error may further attenuate our estimated impacts toward zero and result in an underestimation of the effects of Medicaid benefit design on vaccination coverage.

This study is based on the work of two previous efforts to collect information on state laws. These efforts, funded by CDC, were quite intensive. Unfortunately, no information about changes in cost-sharing arrangements in state Medicaid programs was collected in the time between the two endpoints. This unfortunately places some limitations on the power of the study, as we are unable to exploit associations in year-to-year changes in vaccine coverage and Medicaid cost-sharing design. Further collection of this data would be merited to explore whether states are modifying cost-sharing for grandfathered populations.

The ACA permits states to expand their Medicaid programs to include childless adults whose incomes are 138% of the Federal Poverty Level (FPL) or lower. Prior to the ACA, eligibility limits for this group were often well under 100% FPL. As of April 2016, 31 states and the District of Columbia have expanded their Medicaid programs [25], and an estimated 7 million low-income adults have become eligible for Medicaid as a result [26]. Adults who gain eligibility under Medicaid expansions, most of which took place in 2014—two years after our data stops, will receive coverage for all ACIP-recommended vaccines without cost sharing. Our findings suggest that providing this coverage without cost-sharing could result in higher influenza vaccination coverage among newly-eligible adults. A recent study indicated that few states planned to expand Medicaid benefits for traditionally eligible enrollees to match the benefits that will be provided to new enrollees, even among states that do not currently cover all recommended vaccines without cost-sharing [12]. Medicaid programs in all states may wish to monitor adult vaccination uptake for both groups in their state to observe the effects of benefit design decisions on vaccination coverage.

## 5. Conclusions

This study demonstrated the degree to which vaccination coverage levels are responsive to out-of-pocket costs associated with vaccination services for Medicaid enrollees. We found that influenza vaccination coverage is responsive to various aspects of Medicaid benefit design affecting coverage and cost-sharing. Increased copayments may have implications for vaccine uptake and service utilization, especially among low-income adults, and for disease transmission among the general public. It is important for all states to monitor traditionally-eligible and—where applicable—newly-eligible Medicaid beneficiaries in order to determine the effects of state policy decisions on uptake of recommended vaccines.

## Figures and Tables

**Table 1:** Mean vaccination coverage levels and benefits policy parameters for 2003 and 2012.

Parameter	2003	2012
Imputed Medicaid population: pregnant women	1.0 million	0.7 million
Imputed Medicaid population: parents	13.3 million	10.6 million
Mean influenza vaccination coverage (%) (95% CI)	33.1 (32.7, 33.5)	34.6 (34.3, 34.9)
Mean copayment for vaccination services (USD$)	1.45	1.22
% of respondents in states covering influenza vaccination (number of states)	85.4 (43)	93.7 (49)
% of respondents in states prohibiting cost sharing (number of states)	0 (0)	24.6 (20)

Notes: Influenza vaccination coverage levels are from the 2003 and 2012 Behavioral Risk Factor Surveillance System (BRFSS). State policy attributes are from previously published reports [11,12]. Means and 95% confidence intervals on the means are given for population level variables separately for 2003 and 2012. Copayments are measured in nominal dollars. Each mean was significantly different across years (*p* < 0.01). All means are weighted by the fee-for-service Medicaid population. The number of states (excluding District of Columbia) that allowed cost sharing or covered the influenza vaccination is given for each year in parenthesis after the mean. The final two rows show the percent of respondents affected by the indicated cost-sharing design with the number of states implementing the indicated design in parentheses.

**Table 2:** Difference-in-difference impacts of covering the influenza vaccine on influenza vaccination coverage for adults, 2003 vs. 2012 BRFSS data.

Statistic	All Adults Age 19–64	All Adults Age 50–64	Medicaid Enrolled Pregnant Women Age 19–64	Medicaid Enrolled Parents Age 19–64
	(N = 444,211)	(N = 187,256)	(N = 1335)	(N = 17,255)
Impact on Influenza Vaccination	0.036	0.062	0.047	−0.000
95% CI	(0.022, 0.049)	(0.040, 0.085)	(−0.156, 0.251)	(−0.079, 0.079)
*p*-value	<0.01	<0.01	0.65	0.99
2003 Vaccination Level (%)	28.6	41.7	15.2	19.2

Notes: Influenza vaccination coverage levels are from the 2003 and 2012 BRFSS. State policy attributes are from previously published reports [11,12]. Each coefficient is from a separate regression for the indicated population and policy parameter. Each regression includes controls for state of residence and year.

**Table 3:** Difference-in-difference impacts of prohibiting cost-sharing on influenza vaccination coverage for adults, 2003 vs. 2012 BRFSS data.

Statistic	All Adults Age 19–64	All Adults Age 50–64	Medicaid Enrolled Pregnant Women Age 19–64	Medicaid Enrolled Parents Age 19–64
	(N = 444,211)	(N = 187,256)	(N = 1335)	(N = 17,255)
Impact on Influenza Vaccination	0.002	0.003	0.054	−0.029
95% CI	(−0.007, 0.012)	(−0.013, 0.020)	(−0.123, 0.231)	(−0.088, 0.031)
*p*-value	0.65	0.68	0.55	0.35
2003 Vaccination Level (%)	28.6	41.7	15.2	19.2

Notes: Influenza vaccination coverage levels are from the 2003 and 2012 BRFSS. State policy attributes are from previously published reports [11,12]. Each coefficient is from a separate regression for the indicated population and policy parameter. Each regression includes controls for state of residence and year.

**Table 4:** Difference-in-difference impacts of copayment charges on influenza vaccination coverage for adults, 2003 vs. 2012 BRFSS data.

Statistic	All Adults Age 19–64	All Adults Age 50–64	Medicaid Enrolled Pregnant Women Age 19–64	Medicaid Enrolled Parents Age 19–64
	(N = 299,344)	(N = 134,459)	(N = 813)	(N = 10,813)
Impact on Influenza Vaccination	−0.006	−0.011	−0.062	−0.014
95% CI	(−0.012, −0.001)	(−0.019, −0.002)	(−0.145, 0.022)	(−0.047, 0.018)
*p*-value	0.014	0.014	0.146	0.383
2003 Vaccination Level (%)	29.2	42.2	15.7	18.3

Notes: Influenza vaccination coverage levels are from the 2003 and 2012 BRFSS. State policy attributes are from previously published reports [11,12]. Each coefficient is from a separate regression for the indicated population and policy parameter. Each regression includes controls for state of residence and year.
